# Protocol for a systematic search and critical discourse analysis of research on national HIV pre-exposure prophylaxis programmes among gay, bisexual, and other men who have sex with men

**DOI:** 10.12688/hrbopenres.13841.2

**Published:** 2024-10-25

**Authors:** David Comer, Chris Noone

**Affiliations:** 1 School of Psychology, University of Galway, Galway, H91 EV56, Ireland

**Keywords:** HIV, PrEP, Implementation, gbMSM, sexual health, critical discourse analysis

## Abstract

**Background:**

HIV pre-exposure prophylaxis (PrEP) is a medication that prevents the acquisition of HIV, most commonly taken in the form of a pill. PrEP is an efficacious tool for HIV prevention, including among gay, bisexual, and other men who have sex with men (gbMSM). PrEP is often provided through formal PrEP programmes. Research on these programmes may employ discourses shaped by heteronormativity and homophobia. Given that expert language influences how HIV prevention is understood and delivered, problematic discourses in research likely extend into PrEP implementation. This study will use critical discourse analysis (CDA) to explore research on PrEP programme implementation for gbMSM. Within this literature, we will identify interpretive repertoires used to discuss gbMSM; the subject positions afforded to gbMSM; and the implications of these interpretive repertoires and subject positions for gbMSM engaging with HIV PrEP programmes.

**Methods:**

Systematic search methods identified relevant articles through timebound searching (2012–2023) in ProQuest ASSIA, EBSCOhost PsycInfo, OVID Medline, OVID Embase, and EBSCOhost CINAHL, with forward and backward citation searching of included studies. Grey literature will be identified through ProQuest and Google Scholar. Screening will be conducted by two independent reviewers, who will conduct random partial double screening for titles and abstracts and for all full text articles. Data will be analysed and synthesised using CDA informed by critical realism (CR). CDA focuses on relationships between language and power, including how language enables inequality. The analytic process will explore the background of included studies, identify themes, and analyse external and internal relations in included studies.

**Conclusions:**

Highlighting issues with discourses in PrEP implementation may enhance reflective engagement with assumptions underlying this research, preventing further stigmatisation of gbMSM’s sexual and protective practices. As PrEP programmes become more common, diverse and inclusive perspectives in PrEP programme research may inform interventions that enhance their acceptability and implementation.

## Introduction

### Background

HIV pre-exposure prophylaxis (PrEP) is a medication taken by people without HIV that can prevent the transmission of HIV. PrEP is effective in preventing HIV acquisition from sexual partners living with HIV, and acquisition through sharing of injection equipment, when taken in an effective manner ( Fitch *et al.*, 2022). Consequently, it is an attractive option for gay, bisexual, and other men who have sex with men (gbMSM) seeking to optimise their safety and pleasure during sex. gbMSM are disproportionately affected by HIV; in 2021, 68% of new HIV diagnoses in western/central Europe and North America occurred in gbMSM ( Joint United Nations Programme on HIV/ & AIDS, 2022). The provision of PrEP to groups affected by HIV is often conducted through formal PrEP programmes. PrEP is most commonly delivered via an oral pill, taken daily or during periods when HIV acquisition is most likely. In addition to oral pills, PrEP is available via long-acting injectable (LAI) or through vaginal ring in some settings, as recommended by the World Health Organisation (2022). PrEP programmes should adequately identify those affected by HIV, offer those individuals PrEP, and provide ongoing support for PrEP use as needed ( WHO, 2018).

Variations exist in how these programmes are delivered, but they often include testing for HIV and other sexually transmitted infections (STIs), adherence support, and additional support via counselling and educational resources on sexual health ( Centre for Disease Control and Prevention: US Public Health Service, 2021; ECDC, 2021). Many PrEP programmes offer PrEP medication at free or reduced rates. In some places, such as the United States, financing PrEP is more complex, especially for those without health insurance who may rely on a combination of federal and state supports and manufacturer assistance programmes ( Killelea *et al*., 2022). However, clinical guidelines for PrEP programmes are broadly similar across settings in that they emphasise support for those prescribed PrEP beyond provision of medication.

The clinical criteria for PrEP programme access often require prospective PrEP users to report sexual behaviours or other factors that are perceived as signalling them as being more likely to acquire HIV ( Cornelisse *et al.*, 2018). This risk-focused framing is dominant in HIV prevention research and practice; it has been tied to the increasingly prevalent biomedicalisation of HIV prevention, especially following the emergence of PrEP as a viable prophylactic option ( Race, 2021). This is despite a complex history of HIV prevention that extends beyond biomedical treatment (e.g., risk reduction strategies; Davis, 2021). Furthermore, the success of any biomedical approach to HIV prevention requires attending to social inequalities and structural factors that determine vulnerability to HIV and dictate the accessibility of prevention methods, testing and treatment ( Nguyen *et al.*, 2011). The link between biomedical interventions and social context suggests that HIV interventions must be embedded into the everyday lives of prospective users ( Kippax & Stephenson, 2012), with social and political conditions that maximise autonomy in access to and use of PrEP programmes among gbMSM ( Seckinelgin, 2021). Equitable and inclusive PrEP programme implementation requires recognising the influence discourses about PrEP have on services and service users.

Recent research has examined prevalent discourse on PrEP in different contexts. Hascher *et al.* (2023) explored the influence of a risk-based narrative on the discourses employed by sexual minority men through sexual encounters, with some focus on PrEP. They generated themes centred on the alienation of sexual minority men through risk discourse; stigmatising framings of risks manifesting through slut-shaming in the community; and the enactment and embodiment of risk discourse in relation to sexual positioning, condom use, and perceptions of PrEP. Collins (2022) investigated the concept of risk in relation to PrEP when discussed in news coverage in the US, UK, and Ireland, recognising that different conceptualisations of risk aligned with particular political or ideological aims for media outlets. Mowlabocus (2020) focused on the development of discourses of PrEP in the UK press, as framing of PrEP over time shifted from a miraculous new treatment to a promiscuity-promoting pill that drew resources from other groups considered more deserving. Spieldenner (2016) outlines discourses around PrEP within communities of gbMSM, and how discourses around “PrEP whores”, dirty/clean binaries, the loss of condoms, and the inner whore are navigated and interconnected. More recently, Lewis & Melendez-Torres (2023) identified discursive themes from TikTok videos on PrEP that included a theme of poor healthcare and education for gbMSM using PrEP, as well as the stigmatisation of: HIV as a gay disease; gay men as untrustworthy and unsafe; and PrEP as increasing “unsafe” sexual practices. These various analyses of PrEP discourse identify prevailing narratives that are shaped by heteronormativity and homophobia. Many of these themes were prevalent in discussions of HIV long prior to the advent of PrEP but may have been reconfigured in relation to newer HIV prevention methods. Researchers have been critiquing the negative impact of HIV discourses surrounding promiscuity ( Bolton, 1992) and responsibility ( Strebel & Lindegger, 1998) for over 30 years. Recognising what these problematic discourses achieve and the opportunities for the accessibility of PrEP that they close down allows HIV prevention efforts to better align with best practice in a non-stigmatising manner, enhancing inclusion at clinical and policy level. This raises questions about the kinds of discourses that are drawn upon by those researching and implementing PrEP programmes and what the implications of these discourses might be for services and service users.

### The current study

This study seeks to apply Critical Discourse Analysis (CDA) informed by a critical realist perspective, to literature describing research on PrEP programme implementation. Critical realism (CR) examines complex, open systems, and promotes justice and equality through inquiry. It recognises social life as tied to politics, ethics, and morality – and that these matters influence research, especially when unacknowledged ( Alderson, 2021). Critical discourse analysis understands language and discourse as routes through which power is exercised – institutional power embedded in language influences the social construction of gbMSM. These men are then acted upon through research, policy, and healthcare initiatives such as PrEP programmes ( Carter *et al.*, 2022).

We do not aim to identify truth within the literature or to appraise strength of evidence, but rather explore what expert language reveals about conceptions of gbMSM who use PrEP programmes thereby aiming to highlight potentially problematic discourses in existing research on PrEP programmes for gbMSM. We will analyse the interpretive repertoires that researchers draw on when discussing HIV PrEP use by gbMSM and the subject positions they afford to gbMSM who use PrEP. Experts in a given field have access to both expert and non-expert discourses, while non-experts may only access non-expert discourses; this contributes to a power dynamic whereby experts and institutions hold power over lay people ( Bloor & Bloor, 2007). Those engaging in research on PrEP are often considered experts –expert language holds particular weight in shaping discourse and empirical “evidence”. Research can be seen as “constructing and organising the terms in which particular social issues are understood” ( Schroeder *et al.*, 2022, p. 2). Previously, scholars have argued that the evidence produced and language used in research on substance use among gbMSM exacerbates stigmatisation based on identity ( Race *et al.*, 2016; Schroeder *et al.*, 2022). Expert discussion of gbMSM’s use of PrEP programmes validates the use of particular language or narratives in public discussion; shapes political and health service approaches to HIV prevention and PrEP delivery; and may constrain how service-users and providers conceptualise their role in HIV prevention. This research seeks to make future research more inclusive, prevent continued stigmatisation of the sexual choices made by gbMSM, and contribute to more effective approaches to PrEP research.

The research questions seek to identify interpretive repertoires and subject positions in existing literature. We understand interpretive repertoires as being the collection of terms, descriptions, systems, and building blocks used to manufacture versions of actions or events ( Schiffrin *et al.*, 2001); concepts such as homophobia are not static, but instead comprise flexible and varying contents that may be contradictory or inconsistent. The interpretive repertoire is a line of argument through which sense is made and actions or events are understood ( Wetherell, 1998). Using particular interpretive repertoires then frames the resources and options available to respond to such actions or events.

We understand subject positions as being the ascription of certain rights, duties, or obligations in particular situations; this position dictates the role an individual may fulfil and governs what someone in that position can say or do ( Harré, 2005). The position(s) afforded to a subject are unfixed and respond to social context and the interpretive repertoires at play, changing and overlapping as discourse develops ( Wetherell, 1998).

An example, adapted from Golden and Pomerantz (2015), could describe an interpretive repertoire for sexual health whereby testing is understood as initiating a devastating chain of events. Consequently, those who are unaware of their sexual health status are afforded a subject position in which they can continue with their life as usual, where the existence of an STI has not materialised; by contrast, those who know they have a particular STI are burdened with the actualised realities of responding to that condition.

The research seeks to answer the following research questions:

1. What interpretive repertoires are used to discuss gbMSM in literature on the implementation of national HIV PrEP programmes?
2. What subject positions are made available to gbMSM who are considering, on, or have discontinued PrEP use?
3. What are the implications of these interpretive repertoires and subject positions for how gbMSM are understood and catered to in the implementation of national HIV PrEP programmes?

## Methods

The present systematic search and discourse analysis protocol was registered on the Open Science Framework (OSF) and is available at https://doi.org/10.17605/OSF.IO/JWFUA. The development of this protocol was conducted in line with the Preferred Reporting Items for Systematic Review and Meta-Analysis Protocols (PRISMA-P) guidelines ( Moher *et al.*, 2015; Shamseer *et al.*, 2015). The PRISMA-P checklist with labelling for the location of each item within this study can be found at the associated OSF project page ( Comer, 2023). Required amendments to this protocol will be conducted and reported in line with the PRISMA statement ( Page *et al.*, 2021).

### Eligibility criteria

We will include studies published from 2012 to 2023, as this is the period during which HIV PrEP has been available. Eligibility for the study is informed by the SPIDER tool for mixed-methods systematic screening ( Cooke *et al.*, 2012)

Following the completion of title and abstract screening, we further restricted our eligibility criteria due to the unanticipated breadth of included studies at this stage. Changes to eligibility criteria introduced prior to full-text screening are intended to make screening more feasible and further isolate discourse surrounding gbMSM using PrEP programmes. The most notable of these changes restricts the inclusion of articles to only those evaluating national programmes.

***Sample.*** Included studies will include research on the implementation of PrEP programmes that include gbMSM, including trans gbMSM. Studies that also include other populations will be included, with only sections where gbMSM are discussed being coded for analysis. We will also include research covering the perspectives of staff or policymakers on the implementation of PrEP programmes that include gbMSM.

For full text screening, included studies will include gbMSM, including trans gbMSM, where gbMSM comprise greater than 50% of the study sample. Studies where this information is not available, but samples are likely to include a majority of gbMSM will also be included. We will also include research covering the perspectives of staff or policymakers.

***Phenomenon of interest.*** Studies will discuss the implementation, use of, and access to pill-based HIV PrEP programmes by gbMSM. “PrEP programme” refers to the formal provision of PrEP through a service, including both free and fee-based delivery through community, public service, or private modes of delivery. To be considered a programme, the delivery of PrEP must include some additional support or component beyond solely the provision of medication for PrEP. PrEP pills are the most common PrEP modality for gbMSM, and much of the research on PrEP implementation focuses on PrEP pills. Given the differences between PrEP pills and other PrEP modalities, and the relative recency of the introduction of new types of PrEP, discourses focusing on PrEP delivered via pill may have key differences compared to discourses on other PrEP modalities. Included studies will be limited to those providing PrEP via pills. We will include studies of PrEP programmes operated in the context of PrEP implementation and demonstration projects, where that study attempts to demonstrate a model for PrEP delivery to be embedded into service as usual. Studies that report on the delivery of PrEP in the context of a clinical trial or trial extensions will be excluded owing to differences between how PrEP is delivered within trials and PrEP implementation within the context of standard delivery via a health system.

For full text screening, studies will discuss the implementation of national pill-based HIV PrEP programmes only. We will not include studies of PrEP programmes operated in the context demonstration projects. Only studies reporting on national PrEP delivery through a formalised programme will be included. This includes studies which explicitly or implicitly suggest a national, rather than local or regional, focus of their research. Studies focusing on multiple national programmes will be included. Studies focused on attitudes of those who may access PrEP programmes or other actors will be included only if those attitudes are specifically related to the effective implementation of national PrEP programmes.

***Design.*** Studies of any design will be included. This includes data collection through surveys, interviews, or any other method of primary data collection. This also includes research focusing on secondary data, such as reviews. Data analysis may include descriptive or inferential analyses of quantitative data, or any analyses of interviews or other qualitative data. Research not reporting primary or secondary research will be excluded (e.g., commentaries, letters to the editor, and opinion papers). Eligible research identified via conference abstracts or presentations will be included where an associated textual document (e.g., published paper, report) can be identified.

***Evaluation.*** Included studies must examine the experiences or perceptions of gbMSM in relation to the implementation of PrEP programmes, including but not limited to their experiences of navigating programmes or their perceptions of a programme or the experiences or perceptions of those involved, at any level, in the implementation of PrEP programmes that include gbMSM as service users.

For full text screening, included studies must examine the experiences or perceptions of gbMSM in relation to the implementation of national PrEP programmes where the implementation of a PrEP programme is the specific research focus.

***Research type.*** Qualitative, quantitative, and mixed-methods studies will be eligible for inclusion. The research need not necessarily have taken place at an academic institution or been published in an academic journal. Relevant grey literature, such as reports from health services or community organisations, will be included.

### Exclusion criteria

Studies published in a language other than English will be excluded; discourse analysis focuses on language and as such translated versions of non-English research would not authentically represent the writing of the original researchers. Studies not meeting the SPIDER inclusion criteria will be excluded. We will exclude studies that do not include gbMSM as participants or do not include the views of relevant actors on PrEP implementation for gbMSM. Studies that study PrEP modalities other than the PrEP pill will be excluded, as will studies that only study PrEP as a pill or do not include information relevant to PrEP programmes. Clinical trials and studies not reporting primary or secondary research (e.g., letters to the editor and opinions) will be excluded. Studies that do not evaluate the perspectives of gbMSM or other actors in relation to PrEP programmes will be excluded.

For full text screening, studies that report not including a sample of more than 50% gbMSM or do not include the views of relevant actors on PrEP implementation for gbMSM will be excluded. Studies that do not specifically focus on national PrEP programme implementation, studies that include attitudes towards PrEP not specifically focused on effective PrEP implementation will be excluded.

### Sources of information

Searching will be conducted through five electronic databases informed by consultation with a University of Galway librarian and previous reviews of PrEP in gbMSM (e.g., Hillis *et al.* (2020); Sewell *et al.* (2022); Zhang *et al.* (2022)). These databases are ProQuest ASSIA, EBSCOhost PsycInfo, OVID MEDLINE, OVID Embase and EBSCOhost CINAHL. Forward and backward citation searching will be conducted in those studies deemed eligible for inclusion using citationchaser ( Haddaway *et al.*, 2021). Where a study has not been excluded through title and abstract screening and full text is not available, the study authors will be contacted to request the full-text version. Where an author has not responded within 15 working days (3 weeks), the study will be excluded. Our search for grey literature will be partially informed by the recommendations of Godin *et al.* (2015). This strategy recommends searching grey literature databases; custom google search engines for relevant government and organisational sites; targeting relevant websites; and contacting experts in the field. However, other have suggested that the efficiency of different approaches to grey literature searching are unclear, and note significant pragmatic considerations around grey literature searching ( Adams *et al.*, 2016). In addition, there is very limited existing guidance on the development of custom Google search engines for grey literature searching. Consequently, we will limit our grey literature search to a broad search in ProQuest (as detailed below) and a detailed search of Google Scholar following the guidance of Haddaway *et al.* (2015). This guidance suggests grey literature is more easily identified in Google Scholar via title only searches, and that researchers should search the first 50 pages, or 1000 records, of results (rather than a previously established norm of 10 pages).

### Search strategy

A search strategy developed with a librarian informed by previous research can be seen in Table 1. This search strategy will also be adapted to ProQuest in order to identify grey literature. ProQuest searching will be limited to the source types using the advanced search function: Annual Report, Article, Conference, Conference Paper, Conference Proceeding, Country Report, Dissertation/Thesis, Evidence Based Healthcare, General Information, Government & Official Document, Instructional Material/Guideline, Reference Document, Report, Research Topic, Standard, Technical Report, and Working Paper/Pre-print. The search will be limited to “Anywhere except full text – NOFT” and to English language studies.

The search in google scholar will be limited to title searches of the first 50 pages, with the search string entered directly into the search field using the Boolean operator “OR” and parentheses to separate search terms and concept blocks. All searches will be timebound from 2012–2023.

**Table 1.** Sample search strategy for this study.

Sample Search Strategy for OVID Medline
exp Gays/ OR exp Bisexuals/ OR exp homosexuals/ OR exp Homosexuality, Male/ OR MSM.tw. OR gbMSM.tw. OR men who have sex with men.tw. OR gay*.tw. OR homosexual*.tw. OR bisexual*.tw.
AND
exp pre-exposure prophylaxis/ OR pre-exposure prophylaxis.tw. OR preexposure prophylaxis.tw. OR pre-exposure chemoprophylaxis.tw OR preexposure chemoprophylaxis.tw. OR PrEP.tw. OR truvada.tw. OR tenofovir.tw. OR emtricitabine.tw. OR TDF.tw. OR FTC.tw.
AND
exp HIV/ OR exp Acquired Immunodeficiency Syndrome/ OR HIV.tw. OR human immunodeficiency virus.tw. OR AIDS.tw.
AND
Implement*.tw. OR Program*.tw. OR Deliver*.tw. OR service*.tw. OR train*.tw. OR education*.tw. OR linkage.tw. OR provid*.tw. OR utili#ation.tw

### Study records

***Data management.*** Results of database searching will be exported to Zotero ( Roy Rosenzweig Center for History and New Media, 2023), where duplicates will be removed. Screening will be conducted using Covidence ( Veritas Health Innovation, 2023). We will also extract bibliographic data and study characteristics using Covidence. The full text documents reporting the included studies will be imported to NVivo ( QSR International Pty Ltd., 2020) to facilitate coding and theme generation for the CDA.

***Selection & data collection processes.*** The eligibility and exclusion criteria were developed by DC and CN. Article screening was conducted by two independent reviewers for 37% of included studies; we continued screening papers until we were confident in our application of the inclusion and exclusion criteria. The remaining texts were screened by a single reviewer. All full text articles will be double screened. Where disagreements arise at any stage, a third reviewer will be consulted to make a decision on article inclusion. The results of screening will be compiled in a PRISMA flow diagram with reasons for exclusion documented.

***Data items.*** Given the nature of this study, the entire contents of published articles, including the introduction and discussion, are relevant to answering the research questions and will be coded as described in our description of the analytic approach below. Specific data items for extraction includes bibliographic information (title, authors, publication year and journal, funding, country of origin) and study characteristics (aims/purpose, time of data collection, description of the PrEP programme, sample size, theoretical framework (if applicable), and key findings).

***Study appraisals.*** Given this study seeks to explore discourse used when discussing gbMSM using PrEP programmes rather than study results, the quality of research is not relevant to the research questions. As such, risk of bias assessments, critical appraisals or assessments of confidence in cumulative evidence will not be conducted.

### Analysis

***Critical realism and critical discourse analysis.*** Data will be analysed and synthesised using a critical discourse analysis informed by a critical realist perspective. Critical realism (CR) seeks to explore the real, the actual, and the empirical. In this context, “real” refers to objects, structures, and their causal powers. “Actual” refers to events and process that occur when the real is enacted. “Empirical” refers to how events are experienced by actors ( Joseph & Roberts, 2004; Sims-Schouten & Riley, 2019). CR uses retroductive reasoning to make non-linear claims about the structures and mechanisms that influence individual experience ( Sims-Schouten & Riley, 2019), positing factors or circumstances without which an outcome or concept could not occur ( Meyer & Lunnay, 2013). It examines complex, open systems and promotes justice and equality through this enquiry. Critical discourse analysis (CDA) understands language and text as a social practice ( Cascio & Yomtovian, 2013); according to Fairclough ( Fairclough, 2017, p. 35), CDA “combines critique of discourse and explanation of how discourse figures in existing social reality as a basis for action to change reality”. In particular, CDA takes interest in the relationship between language and power ( Wodak & Meyer, 2001), including how that language is used to enable or reproduce abuse, injustice, or inequality. CDA places particular emphasis on power within linguistics, including how that language may control action or cognition ( Mullet, 2018)

A critical realist approach to critical discourse analysis posits that while meaning is constructed through interaction, elements outside of discourse (extra-discursive elements) also influence that meaning; some discourses are more easily accessible owing to underlying extra-discursive factors relating to, for example, biochemical facts or the organisation of physical material in space ( Sims-Schouten *et al.*, 2007). Sim-Schouten *et al.* (2007) describe three extra-discursive factors: embodiment (relating to the body and its interactive functions), materiality (relating to physical matter), and institutions (which exist outside of the social construction of their policies and impact) ( Sims-Schouten *et al.*, 2007). A CR approach to CDA looks to explore power enacted through discourse, with the possibilities of that discourse shaped by constraints in the material world.

***Analytic process.*** The analytic framework can be seen in Table 2.

**Table 2.** Analytical framework for CDA as applied to the current research on PrEP programmes for gbMSM (adapted from Mullet, 2018).

Stage of analysis		Description	Example within the current research
1	Select the discourse	Select a discourse related to injustice or inequality in society	Discourses employed in research on the implementation of PrEP programmes for gbMSM.
2	Locate and prepare data sources	Select data sources (texts) and prepare the data for analysis	Systematic search of databases and grey literature relating to the implementation of PrEP programmes for gbMSM
3	Explore the background of each text	Examine the social and historical context and producers of each text	Exploring the historical and social context through which HIV prevention methods have been developed within communities of gbMSM, healthcare providers, and policymakers.
4	Code texts and identify overarching themes	Identify the major themes and subthemes using choice of qualitative coding methods	Identify key elements of critical discourse analysis (e.g., interpretive repertoires drawn upon by researchers and subject positions afforded to gbMSM) to be coded inductively following familiarisation with text.
5	Analyse the external relations in the texts (interdiscursivity)	Examine social relations that control the production of the text; in addition, examine the reciprocal relations (how the texts affect social practices and structures). How do social practices inform the arguments in the text? How does the text in turn influence social practices?	Exploring dominant social practices and norms (e.g., PrEP use as signifying promiscuity), social structures (e.g., the manner and context in which PrEP programmes are implemented).
6	Analyse the internal relations in the texts	Examine the language for indications of the aims of the texts (what the texts set out to accomplish), representations (e.g., representations of social context, events, and actors), and the speaker’s positionality.	Exploring the use of vocabulary, grammar, and linguistic devices to understand discursive accomplishments, interpretive repertoires, and subject positions.
7	Interpret the data	Interpret the meanings of major themes, external relations, and internal relations identified in stages 4, 5, and 6.	Generating broad themes that encompass the interactions between key elements of discourse, dominant social practices and structures, and interpretive repertoires/subject positions to explain how these factors influence the delivery and implementation of PrEP programmes for gbMSM.

CDA= critical discourse analysis; HIV= human immunodeficiency virus; PrEP= HIV pre-exposure prophylaxis; gbMSM= gay, bisexual, and other men who have sex with men.

DC will lead analysis and synthesis in collaboration with CN throughout. We will draw on the analytical framework for CDA described by Mullet (2018). This framework requires the selection of discourse related to inequality and the locating of texts, the processes and justification for which are described in this protocol. Following this, we will explore the background of texts (e.g., intended audience, genre, purpose, and the historical context in which the text was written). We intend to then identify external relations to understand how the text has been produced in context and how the text may subsequently inform that context (e.g., by looking at social practices and norms, social structures). In addition, internal relations will be explored to identify the aims of the text, power relations, and how the speaker and others are represented in the text (e.g., by looking at leading statements, layout, choice of quoted material, vocabulary, contrast, or statements of absolute truth).

Our analysis will also examine presuppositions (knowledge assumed to be known by readers) and implicatures (meanings not explicitly stated but nonetheless evident) ( Bloor & Bloor, 2007). We will also specifically code for interpretive repertoires, through exploring what kind of reality is being constructed and what other realities are resisted as a consequence, with a focus on power and on human agency. Following this, we will examine and code the subject positions made available through the interpretive repertoires at play ( Locke & Budds, 2020). This will include understanding how positions are actualised with particular focus on “reference” (i.e., how different groups and social roles are referred to and have their identities realised) ( Bloor & Bloor, 2007). Analysis will also assess the aims and accomplishments of particular discursive choices and techniques; the analysis of this will draw on some of the tools of discourse analysis outlined by Gee (2010). Finally, we will interpret the meaning of the major themes identified through the coding of these components (internal and external relations, subject positions, interpretive repertoires, discursive accomplishments).

Included studies will be read iteratively in chronological order (least to most recent date of publication) with the researchers coding them as above, taking notes and engaging in discussion about the language in the text. Given that CDA requires scrutiny of that which is omitted, and coding for absences is not possible, detailed observational notetaking will be conducted throughout the coding process. Codes and notes will be assessed and discussed between authors to generate broader understandings of included texts. Results will be summarised thematically, identifying discursive tools at play and how these interrelate to form key discursive concepts across texts.

### Reflexivity

Qualitative research is a subjective process ( Braun & Clarke, 2013) and as such reflexivity will be required throughout this study. Both DC and CN are social science researchers with backgrounds in health psychology. DC is a PhD student, while CN is a psychology lecturer and researcher with an interest in the experiences of health and healthcare of gbMSM. Both researchers are white, queer men living in Ireland. Throughout the research process, authors will reflect on their positions and how their preconceptions influence the research process through open discussion and a reflexive journal kept by DC.

## Study status

Searching for this study has been completed. We identified 9578 articles through our database search and conducted title and abstract screening on 4487 articles following deduplication. Full-text screening is ongoing.

## Discussion

As noted above, while research has identified various discourses in relation to the use of PrEP, how these or other discourses operate in the context of research on the implementation of PrEP programmes has not been studied. It is important to study discourse within PrEP implementation research because while sexual health strategies often describe sexual health as comprising pleasurable and safe sexual experiences, this is often neglected in practice ( Kutner *et al.*, 2021). Many mass media campaigns ( French *et al.*, 2014) and behavioural interventions ( Lorimer *et al.*, 2013) take a myopic lens to HIV that focuses on reducing or avoiding acquisition rather than promoting wellbeing or holistic experiences of sex. Furthermore, policy and practice guidelines frequently fail to draw on aspects of PrEP outside of HIV avoidance as a method for improving acceptability or uptake ( Kutner *et al.*, 2021). This is despite evidence that sexual pleasure motivates sexual behaviour, and as such has potential to be leveraged in PrEP promotion. gbMSM on PrEP have variously been found to report lower HIV-related anxiety, greater intimacy, improvements in sex life quality, and less anxiety around the barrier to pleasure presented by condoms ( Kutner *et al.*, 2021). In addition, reduced worry and anxiety around sex generally has been documented as a motivator to PrEP use ( Gredig *et al.*, 2016).

The biomedicalisation of PrEP access and use also biomedicalises the sexual experiences of gbMSM ( Young *et al.*, 2016). This is potentially problematic given that gbMSM availing of PrEP are not just revealing an awareness of biomedical risk, but also a holistic understanding of PrEP and how it may apply to their personal and social experience. They are expressing a desire to have sex, or to have sex in particular ways ( Seckinelgin, 2021). Risk-based approaches not only neglect to reflect the full experience of gbMSM, but potentially stigmatise gbMSM based on their choice to take or not take PrEP. Prevailing narratives that position PrEP programme users as risky contribute to stigmatisation of PrEP use, whereby gbMSM who take PrEP are viewed as promiscuous ( Dubov *et al.*, 2018) – a view rooted in sex negativity. Alternatively, those who opt not to avail of PrEP may be criticised for failing to take protective action for the benefit of their community. Furthermore, the new sexual opportunities afforded to gbMSM through PrEP (for example, condomless sex) also open opportunity for the policing of behaviour of gbMSM ( Bernays *et al.*, 2021).

This positioning of gbMSM as being inherently risky or vulnerable to disease is damaging to gbMSM while also undermining a holistic approach to HIV prevention. Understanding how gbMSM are positioned by discourses within PrEP implementation research may provide important insights into the accessibility of PrEP programmes. The trajectory of the biomedicalisation of HIV is likely to be affected by new PrEP modalities, such as LAI PrEP. Understanding how discourses surrounding PrEP pills have affected implementation is also relevant to informing the ongoing rollout of new prophylactics. Some recent efforts have recognised the need for moving beyond PrEP biomedicalisation through holistic PrEP services (e.g., differentiated service delivery ( World Health Organisation, 2022)). However, service reconfiguration to support improved models of care depends on evidence that recognises and respects the needs of service users.

In sum, discourses around PrEP in select mediums outside of scientific research have been explored; however, researchers have not yet looked inwardly at the role of potentially problematic PrEP discourses in implementation research. Expert language shapes the way social issues are understood ( Schroeder *et al.*, 2022) and so expert conceptualisations of gbMSM and PrEP use have considerable influence on how HIV prevention is understood and delivered. Problematic discourses in research are likely to be extended into programme implementation and social understandings of PrEP, and these discourses may fail to recognise the diverse needs and experiences of gbMSM and ultimately contribute to reduced effectiveness in PrEP programme implementation. As these programmes are becoming more common globally, diversifying perspectives in research that informs these programmes may facilitate the design of programmes with greater acceptability to potential users and ultimately enhance implementation.

## Data Availability

No data are associated with this article. Open Science Framework: PRISMA-P checklist for ‘Protocol for a systematic review and critical discourse analysis of research on HIV pre-exposure prophylaxis programme use among gay, bisexual, and other men who have sex with men’. https://doi.org/10.17605/OSF.IO/735P4 ( Comer, 2023).
