# Supplementary material for: Protocol for a systematic search and critical discourse analysis of research on national HIV pre-exposure prophylaxis programmes among gay, bisexual, and other men who have sex with men
Source: HRB Open Res. 2024 Oct 25;7:12. Originally published 2024 Feb 29. [Version 2] doi: 10.12688/hrbopenres.13841.2 (PMC11532903; doi:10.12688/hrbopenres.13841.2)
Supplement: Peer review history

# Reviewer report: Reviewer response for version 2

**DOI:** 10.21956/hrbopenres.15286.r42965

**Published:** 2024-10-31

**Authors:** Daniel Hughes

**Affiliations:** 1 Medical College of Wisconsin, Milwaukee, USA

I would consider in the article that addressing gbMSM as more susceptible to STIs is not intrinsically problematic, but rather could be used to positive or negative effect depending on intentions and context. Certainly, a physician making different recommendations for STI screening to a sexually active gay man as compared to a straight man with one partner seems reasonable--and in fact potentially intended to protect the health of the hypothetical gay man.

 Also, in marketing PrEP there are limited resources. Focusing on gay men or other subgroups may prove to be warranted based on limitations on funding, greater uptake by certain populations, or other factors. 

 It would also be useful to consider how gbMSM themselves view current targeting of PrEP campaigns -- positively? negatively? otherwise? 

 Lastly, PrEP uptake has been relatively high among gbMSM in many countries. What have been the successes, alongside failures or deficits, in current approaches?

Is the study design appropriate for the research question?

Yes

Is the rationale for, and objectives of, the study clearly described?

Yes

Are sufficient details of the methods provided to allow replication by others?

Yes

Are the datasets clearly presented in a useable and accessible format?

Yes

Reviewer Expertise:

HIV; PrEP; mpox; gay men

I confirm that I have read this submission and believe that I have an appropriate level of expertise to confirm that it is of an acceptable scientific standard, however I have significant reservations, as outlined above.

# Reviewer report: Reviewer response for version 2

**DOI:** 10.21956/hrbopenres.15286.r42938

**Published:** 2024-10-31

**Authors:** Robin Schaefer, Ekene Osakwe

**Affiliations:** 1 World Health Organization, Geneva, Switzerland; 2 Forum for Collaborative Research, University of California, Berkeley, USA

I thank the authors for providing a comprehensive response to our comments. All of our feedback has been addressed, and we have no further comments.

Is the study design appropriate for the research question?

Yes

Is the rationale for, and objectives of, the study clearly described?

Yes

Are sufficient details of the methods provided to allow replication by others?

Partly

Are the datasets clearly presented in a useable and accessible format?

Not applicable

Reviewer Expertise:

HIV prevention, public health, PrEP, regulatory science; I am not an expert in critical discourse analysis or critical realism, so I am not able to specifically comment on these approaches.

We confirm that we have read this submission and believe that we have an appropriate level of expertise to confirm that it is of an acceptable scientific standard.

# Reviewer report: Reviewer response for version 1

**DOI:** 10.21956/hrbopenres.15165.r41134

**Published:** 2024-07-16

**Authors:** Robin Schaefer, Ekene Osakwe

**Affiliations:** 1 World Health Organization, Geneva, Switzerland; 2 Forum for Collaborative Research, University of California, Berkeley, USA

We thank the editors for inviting us to review this protocol for an important study. The authors aim to conduct a study that looks “inwardly” on the discourse in research around pre-exposure prophylaxis (PrEP) for HIV prevention among men who have sex with men (MSM). This is an important study given that expert language influences how social issues are conceptualized and understood, so this language may exacerbate and create stigma or, conversely, facilitate access to and use of HIV prevention options. The manuscript is well-written, with a clearly described rationale for the study and clearly described objectives. Below, we provide some suggestions to strengthen the manuscript and the underlying study further.

**Introduction: Background**

- 1 ^st^ paragraph, description of how PrEP programmes are delivered: The description applies largely to European PrEP programmes, while the situation is different in the US. The authors may want to clarify this. The authors may also want to reframe the study beyond North America and Europe, as their study does not restrict studies based on geography.
- Same paragraph, and throughout: I suggest avoiding language around “safe sex” as this can be considered stigmatizing. I suggest just generally speaking about “sexual health”.
- 2 ^nd^ paragraph, 3 ^rd^ sentence: The authors mention non-biomedical risk reduction strategies. The authors may want to provide nuance that there is limited evidence on the effectiveness of these strategies relying on behaviour change.
- 2 ^nd^ paragraph, second half: There seems to be some repetition in the second half of the paragraph, so it could be rephrased in a clearer and more concise way.
- Last paragraph of background: The themes outlined in this paragraph are interesting and of course not new. It may be interesting to add historical context, that similar themes were prevalent earlier in the epidemic, before the availability of PrEP.

**Introduction: Current Study**

- 1 ^st^ paragraph, last sentence: The last sentence may be easier to follow if split into two. Also, please check the use of “who” in the sentence.
- 2 ^nd^ paragraph, top of page 6: Similarly, the sentence starting with “Those engaging in research…” may be clearer if divided into two.
- 2 ^nd^ paragraph, last sentence: Check grammar (“makes”)
- 5 ^th^ paragraph, last sentence: Check sentence (“as STI”)

**Methods**

- Eligibility criteria, first paragraph: The authors refer to eligible studies conducted between 2012 and 2023. I would assume this actually refers to the publication year. Please clarify.
- Phenomenon of interest: The authors speak of “PrEP programmes”, but the definition of this is fairly broad. “Programmes” are commonly distinguished from “research studies”, but not all studies in which PrEP is provided may be considered “clinical trials”. There may be studies referred to as “implementation science projects”, which are neither programmes nor clinical trials. The authors should be clear about what they will include.
- Evaluation: It is noted that studies must examine the experience or perceptions of MSM. This study is about what expert language reveals about conceptions about MSM who access PrEP, so I do not think that included studies necessarily need to cover the experience of the PrEP users. For instance, there could be a study focusing on healthcare providers who interact with MSM in a programme, which could be of relevance for this study. I suggest reconsidering this.
- Exclusion criteria: While the inclusion criteria outlined in the previous section implies exclusion criteria, it would be helpful to list them explicitly, rather than just referring to the criteria “outlined above”.
- Analysis: 2 ^nd^ paragraph, 1 ^st^ sentence. Please consider splitting the sentence for clarity.
- Analysis: It may be helpful to reflect on how results will be presented (e.g., how do you aim to summarize your results?).
- Table 2: Please define all acronyms in a table footnote.

**Discussion**

- 2 ^nd^ paragraph: As the authors point out, PrEP has led to a biomedicalization of HIV prevention, and this trend could be exacerbated by newer PrEP products (which require injections) (this could be reflected on). However, it is also true that there are trends towards differentiated service delivery which adapts services to the needs and preferences of those who could benefit from PrEP. Recent WHO guidance emphasizes this. The authors may want to reflect on these approaches to provide balance.

Is the study design appropriate for the research question?

Yes

Is the rationale for, and objectives of, the study clearly described?

Yes

Are sufficient details of the methods provided to allow replication by others?

Partly

Are the datasets clearly presented in a useable and accessible format?

Not applicable

Reviewer Expertise:

HIV prevention, public health, PrEP, regulatory science; I am not an expert in critical discourse analysis or critical realism, so I am not able to specifically comment on these approaches.

We confirm that we have read this submission and believe that we have an appropriate level of expertise to confirm that it is of an acceptable scientific standard, however we have significant reservations, as outlined above.

# Response

**Published:** 2024-10-22

**Authors:** David Comer

**Affiliations:** School of Psychology, University of Galway, Galway, County Galway, Ireland

We appreciate the input of Drs Schaefer and Osakwe to our paper, and their comments which have helped to refine our protocol. Our responses to their comments are outlined below.

 1. We thank the editors for inviting us to review this protocol for an important study. The authors aim to conduct a study that looks “inwardly” on the discourse in research around pre-exposure prophylaxis (PrEP) for HIV prevention among men who have sex with men (MSM). This is an important study given that expert language influences how social issues are conceptualized and understood, so this language may exacerbate and create stigma or, conversely, facilitate access to and use of HIV prevention options. The manuscript is well-written, with a clearly described rationale for the study and clearly described objectives. Below, we provide some suggestions to strengthen the manuscript and the underlying study further.

 Response: Thank you for your kind comments and interest in our research.

 Introduction: Background

 2. 1 ^st^ paragraph, description of how PrEP programmes are delivered: The description applies largely to European PrEP programmes, while the situation is different in the US. The authors may want to clarify this. The authors may also want to reframe the study beyond North America and Europe, as their study does not restrict studies based on geography.

 Response: In the introduction we have provided clarity on PrEP programmes in the USA and outlined differences, particularly in funding routes, that may alter how PrEP is delivered.

 3. Same paragraph, and throughout: I suggest avoiding language around “safe sex” as this can be considered stigmatizing. I suggest just generally speaking about “sexual health”.

 Response: We have changed the language and removed the reference to “safe sex”.

 4. 2 ^nd^ paragraph, 3 ^rd^ sentence: The authors mention non-biomedical risk reduction strategies. The authors may want to provide nuance that there is limited evidence on the effectiveness of these strategies relying on behaviour change.

 Response: The intention of this section is to provide context for the advent of biomedical risk prevention strategies. We are not advocating for or against the use of non-biomedical risk prevention strategies, but rather making it clear that biomedical methods are a new development in a long history of HIV prevention. New approaches to HIV prevention influence changing perceptions of HIV, and it is important to recognise how these alter or conflict with previous approaches.

 5. 2 ^nd^ paragraph, second half: There seems to be some repetition in the second half of the paragraph, so it could be rephrased in a clearer and more concise way.

 Response: We have altered this paragraph to make it more concise.

 6. Last paragraph of background: The themes outlined in this paragraph are interesting and of course not new. It may be interesting to add historical context, that similar themes were prevalent earlier in the epidemic, before the availability of PrEP.

 Response: We have added some further detail on previous research which outlined similar themes to those discussed in our article.

 Introduction: Current Study

 7. 1 ^st^ paragraph, last sentence: The last sentence may be easier to follow if split into two. Also, please check the use of “who” in the sentence.

 Response: We have altered the sentence structure and grammar to make this sentence clearer.

 8. 2 ^nd^ paragraph, top of page 6: Similarly, the sentence starting with “Those engaging in research…” may be clearer if divided into two.

 Response: We have altered this sentence to make it clearer.

 9. 2 ^nd^ paragraph, last sentence: Check grammar (“makes”)

 Response: We have fixed the grammar in this sentence.

 10. 5 ^th^ paragraph, last sentence: Check sentence (“as STI”)

 Response: We have fixed the grammar in this sentence.

 Methods

 11. Eligibility criteria, first paragraph: The authors refer to eligible studies conducted between 2012 and 2023. I would assume this actually refers to the publication year. Please clarify.

 Response: We have clarified that this range refers to publication year.

 12. Phenomenon of interest: The authors speak of “PrEP programmes”, but the definition of this is fairly broad. “Programmes” are commonly distinguished from “research studies”, but not all studies in which PrEP is provided may be considered “clinical trials”. There may be studies referred to as “implementation science projects”, which are neither programmes nor clinical trials. The authors should be clear about what they will include.

 Response: We have provided further detail in the “Phenomenon of Interest” section to clarify how we understand PrEP programmes and related research.

 13. Evaluation: It is noted that studies must examine the experience or perceptions of MSM. This study is about what expert language reveals about conceptions about MSM who access PrEP, so I do not think that included studies necessarily need to cover the experience of the PrEP users. For instance, there could be a study focusing on healthcare providers who interact with MSM in a programme, which could be of relevance for this study. I suggest reconsidering this.

 Response: The sample section of the paper includes detail which states that the perspectives of other actors on PrEP programme implementation will also be included in the research.

 14. Exclusion criteria: While the inclusion criteria outlined in the previous section implies exclusion criteria, it would be helpful to list them explicitly, rather than just referring to the criteria “outlined above”.

 Response: We have added a new section outlining exclusion criteria for this study.

 15. Analysis: 2 ^nd^ paragraph, 1 ^st^ sentence. Please consider splitting the sentence for clarity.

 Response: We have split this sentence as suggested.

 16. Analysis: It may be helpful to reflect on how results will be presented (e.g., how do you aim to summarize your results?).

 Response: We have provided some additional information on how our results will be presented.

 17. Table 2: Please define all acronyms in a table footnote.

 Response: Added a footnote to the table.

 Discussion

 18. 2 ^nd^ paragraph: As the authors point out, PrEP has led to a biomedicalization of HIV prevention, and this trend could be exacerbated by newer PrEP products (which require injections) (this could be reflected on). However, it is also true that there are trends towards differentiated service delivery which adapts services to the needs and preferences of those who could benefit from PrEP. Recent WHO guidance emphasizes this. The authors may want to reflect on these approaches to provide balance.

 Response: We have provided detail on the provision of DSD and how our research relates to this aim.

# Reviewer report: Reviewer response for version 1

**DOI:** 10.21956/hrbopenres.15165.r39889

**Published:** 2024-06-03

**Authors:** Erin E Cooney

**Affiliations:** 1 Johns Hopkins Bloomberg School of Public Health, Baltimore, USA

This study protocol describes the methods and procedures for conducting a critical discourse analysis on published and grey literature related to PrEP implementation and programming for gbMSM populations. The protocol is very well-written, clear, and provides appropriate methodological detail. I have just a few minor suggestions for the authors consideration:

 The authors state in the abstract: “HIV pre-exposure prophylaxis (PrEP) is a pill that prevents the transmission of HIV from sexual partners living with HIV”. Given the FDA and EMA approvals of non-pill/tablet PrEP modalities (e.g., injectable PrEP and vaginal ring), it is important not to provide a definition of HIV PrEP that excludes other available modalities. It would be more accurate to include a broader definition of HIV PrEP and then to clarify that when “HIV PrEP” is used throughout the protocol, the authors are only referring to HIV PrEP pills unless otherwise specified (if this is true). The authors do not explicitly mention other modalities but given that there are no stated geographic limitations, there likely will be relevant papers focused on implementation of long-acting cabotegravir and perhaps the ring. Will these be included or excluded? I suggest adding a few sentences to the methods to specify and justify this decision. I don’t think vaginal rings would be relevant as I believe the authors are only interested in cisgender men, but again, it would be helpful to explicitly confirm one way or another whether gay, bisexual, or other transgender men who have sex with men would be included. There may be differences in the discourse around each PrEP modality so just something to be aware and plan for to the extent possible.

 It is critical not to imply that PrEP is something exclusively or primarily relevant to gay and bisexual men and other cisgender men who have sex with men. The authors state in the abstract: “… [PrEP] is frequently taken by gay, bisexual, and other men who have sex with men (gbMSM).” It would be better to state that PrEP is an efficacious HIV prevention option for any individual who is determined to be susceptible to HIV acquisition which may include gay, bisexual, and other men who have sex with men.

 Background: The authors state “HIV pre-exposure prophylaxis (PrEP) is a pill taken by people without HIV that can prevent the transmission of HIV from sexual partners living with HIV, when taken in an effective manner”. When broadly defining PrEP, it is important to note that PrEP can prevent transmission via shared injection equipment as well as through sexual transmission. Although injection drug use isn’t the focus of this particular analysis, it is a common misconception that PrEP is only for preventing sexual transmission of HIV so it is important to not perpetuate that misconception by omitting the other common indication for which we have excellent evidence of efficacy.

 Methods: I am curious about the exclusion of papers that report on PrEP clinical trials. Could the authors add 1-2 sentences to explain the rationale for excluding these studies? And where would findings from open label extensions of clinical trials fall (include or exclude)?

 Title and use of “systematic review”: I am curious about the use of the term “systematic review” throughout. To me, systematic review implies a synthesis of the results of relevant studies. In this case, where the results are not being synthesized, might it be better to highlight the critical discourse analysis as primary and then refer to “systematic searching” as part of the data acquisition and preparation for this analysis?

 Overall, this is a very interesting analysis and will make an excellent contribution to the literature. Thank you for the opportunity to review and to provide suggestions on this important work.

Is the study design appropriate for the research question?

Yes

Is the rationale for, and objectives of, the study clearly described?

Yes

Are sufficient details of the methods provided to allow replication by others?

Yes

Are the datasets clearly presented in a useable and accessible format?

Not applicable

Reviewer Expertise:

Areas of expertise include mixed methods research, HIV epidemiology, PrEP implementation, LGBTQ health, and systematic reviews

I confirm that I have read this submission and believe that I have an appropriate level of expertise to confirm that it is of an acceptable scientific standard.

# Response

**Published:** 2024-10-22

**Authors:** David Comer

**Affiliations:** School of Psychology, University of Galway, Galway, County Galway, Ireland

We thank Dr Cooney for her helpful and insightful response which has improved our paper. See below our response to specific comments and detail on the changes made.

  

1. *This study protocol describes the methods and procedures for conducting a critical discourse analysis on published and grey literature related to PrEP implementation and programming for gbMSM populations. The protocol is very well-written, clear, and provides appropriate methodological detail. I have just a few minor suggestions for the authors consideration:*

Response: Thank you for your kind comments.

1. *The authors state in the abstract: “HIV pre-exposure prophylaxis (PrEP) is a pill that prevents the transmission of HIV from sexual partners living with HIV”. Given the FDA and EMA approvals of non-pill/tablet PrEP modalities (e.g., injectable PrEP and vaginal ring), it is important not to provide a definition of HIV PrEP that excludes other available modalities. It would be more accurate to include a broader definition of HIV PrEP and then to clarify that when “HIV PrEP” is used throughout the protocol, the authors are only referring to HIV PrEP pills unless otherwise specified (if this is true). The authors do not explicitly mention other modalities but given that there are no stated geographic limitations, there likely will be relevant papers focused on implementation of long-acting cabotegravir and perhaps the ring. Will these be included or excluded? I suggest adding a few sentences to the methods to specify and justify this decision. I don’t think vaginal rings would be relevant as I believe the authors are only interested in cisgender men, but again, it would be helpful to explicitly confirm one way or another whether gay, bisexual, or other transgender men who have sex with men would be included. There may be differences in the discourse around each PrEP modality so just something to be aware and plan for to the extent possible.*

Response: We have altered our introduction and discussion to include broader definitions of PrEP, with some additional detail on alternative PrEP modalities. In our methods section, we have clarified that our study focuses specifically on PrEP pills and provided the reasoning for this.

1. *It is critical not to imply that PrEP is something exclusively or primarily relevant to gay and bisexual men and other cisgender men who have sex with men. The authors state in the abstract: “… [PrEP] is frequently taken by gay, bisexual, and other men who have sex with men (gbMSM).” It would be better to state that PrEP is an efficacious HIV prevention option for any individual who is determined to be susceptible to HIV acquisition which may include gay, bisexual, and other men who have sex with men.*

Response: We agree with the reviewer and have provided clarity in the abstract and introduction that PrEP is effective for all those relatively likely to acquire HIV, including some gbMSM.

  

1. *Background: The authors state “HIV pre-exposure prophylaxis (PrEP) is a pill taken by people without HIV that can prevent the transmission of HIV from sexual partners living with HIV, when taken in an effective manner”. When broadly defining PrEP, it is important to note that PrEP can prevent transmission via shared injection equipment as well as through sexual transmission. Although injection drug use isn’t the focus of this particular analysis, it is a common misconception that PrEP is only for preventing sexual transmission of HIV so it is important to not perpetuate that misconception by omitting the other common indication for which we have excellent evidence of efficacy.*

Response: We have clarified in our introduction that PrEP is effective for preventing HIV transmission through routes other than sexual contact.

1. *Methods: I am curious about the exclusion of papers that report on PrEP clinical trials. Could the authors add 1-2 sentences to explain the rationale for excluding these studies? And where would findings from open label extensions of clinical trials fall (include or exclude)?*

Response: We have provided additional detail in the methods section (under “Phenomenon of Interest”) to explain the exclusion of clinical trials and trial extensions in this research.

1. Title and use of “systematic review”: I am curious about the use of the term “systematic review” throughout. To me, systematic review implies a synthesis of the results of relevant studies. In this case, where the results are not being synthesized, might it be better to highlight the critical discourse analysis as primary and then refer to “systematic searching” as part of the data acquisition and preparation for this analysis?

Response: Upon reflection, we recognise that describing this study as a Systematic Review may diverge from definitions of “Systematic Review” in literature. As such, we have altered the title and use of the term “review” throughout the paper to clarify our use of systematic search methods within a discourse analysis of research.
